# Trust and interaction design in AI-enabled systems: a systematic literature review

**DOI:** 10.3389/fpsyg.2026.1932655

**Published:** 2026-09-15

**Authors:** Summer L. Abramson, Jhonathan Sora-Cardenas, Pranav Nandakumar, Jonathan E. Bruce, Nagomi Gopinath, Lucas Guarenti Zangari, Pedro Guillermo Feijóo-García

**Affiliations:** College of Computing, Georgia Institute of Technology, Atlanta, GA, United States

**Keywords:** AI-enabled systems, trust anthropomorphism, artificial intelligence (AI), design factors, explainability, human-centered AI, human-centered computing, human-computer interaction (HCI)

## Abstract

As artificial intelligence (AI) becomes increasingly integrated into everyday systems, understanding how users perceive and interact with AI is critical for effective design. This systematic literature review analyzes 1,565 peer-reviewed scholarly articles published post-2023 from IEEE Xplore, ACM Digital Library, Scopus, and Web of Science, ultimately including 33 publications that met the inclusion criteria. Following the Preferred Reporting Items for Systematic reviews and Meta-Analyses (PRISMA) framework, this review examines how user trust in AI-enabled systems is defined and measured, and which interaction design factors influence it. The findings reveal that the literature reflects diverse contributions from authors, institutions, and disciplines, while also employing varied definitions and methodologies, contributing to a fragmented body of work. In addition, this review identifies nine key design factors, including explainability, anthropomorphism, confidence, interface, errors, security, transparency, competence, and perceived control. Overall, greater consensus within the field is needed to support the development of stronger and more cohesive bodies of work for each design factor.

## 1 Introduction

Over the past three years, artificial intelligence (AI) and AI-enabled systems–defined here as systems that augment, enhance, or replace functions of existing technologies–have become increasingly embedded across important areas of everyday life, including healthcare (Patil and Shankar, 2023), autonomous vehicles (Xu et al., 2020), and intelligent tutoring systems (Alam, 2023). This rapid integration has changed not only how users approach and interact with these systems, but also how researchers study those interactions. Concurrent with this shift, the public release of generative AI (GenAI) tools such as ChatGPT, Claude, and Microsoft Copilot have dramatically expanded public awareness of and familiarity with AI in a more general way, with nearly 40% of the U.S. population aged 18-64 now using such tools Bick et al. (2024). This growing familiarity has in turn shaped what users know about, and expect from AI-enabled systems across consequential domains.

Despite this shift, the literature examining how interaction design factors influence trust in AI-enabled systems remains fragmented, characterized by inconsistent conceptualizations of trust, limited empirical evidence across certain contexts, and a reliance on subjective measures of system evaluation (Bach et al., 2024; Mehrotra et al., 2024a). Understanding how interaction design shapes trust is particularly important because it directly informs the development of design strategies that support appropriate and calibrated use of AI-enabled systems. Researchers across disciplines have approached this question using different terminology, measurement strategies, and study designs, making it difficult to synthesize results or develop shared design guidance. This challenge is particularly pressing given that the pace of public AI adoption has outpaced the field's ability to consolidate and interpret emerging findings.

This systematic literature review builds on a prior systematic literature review examining the definitions and measurement strategies used to study trust in AI-enabled systems (Bach et al., 2024). This study extends that work in three ways: (1) first, by analyzing shifts in research approaches and findings in the post-generative-AI era; (2) second, by examining participant demographic distributions in greater depth; and (3) third, by thematically comparing the interaction design factors researchers have chosen to manipulate. Where Bach et al. established the foundational landscape of trust measurement in AI systems, this review asks what has changed, and which design factors the field has focused on as AI has become a fixture of daily life.

Findings in human-computer interaction research are inherently contextual, reflecting the specific experimental setup, population, and variables under investigation (Grassini et al., 2025). Nevertheless, this study seeks to examine whether meaningful patterns emerge across varied contexts and environments. Identifying such patterns could support the development of more generalizable design guidance for AI-enabled systems, particularly design principles that promote appropriate and calibrated trust across different domains and use cases.

The intersection of AI, trust, and interaction design draws researchers from multiple disciplines, and as a result the literature has evolved in different directions, often with inconsistent definitions, varied methodologies, and findings that are difficult to compare directly. A systematic review is well suited to synthesizing this landscape, particularly at a moment when publicly accessible AI systems are expanding rapidly. For this purpose, this review addresses three research questions:

1. RQ1: How have disciplinary and institutional trends shaped human-computer interaction (HCI) research on user trust in AI-enabled systems, and how has this focus evolved in recent years as publicly accessible AI systems have expanded?
2. RQ2: What definitions and measurement strategies, such as trust evaluation, systems analyzed, and populations, are used to evaluate user trust in AI-enabled systems?
3. RQ3: What interaction design factors have been shown to affect user trust in AI-enabled systems?

Together, these questions allow us to examine the landscape of how trust is studied, the approaches in which it's evaluated, and what design factors influence it.

### 1.1 Background

#### 1.1.1 Defining trust

Trust has been conceptualized in multiple ways across disciplines and over time, reflecting its inherently complex and context-dependent nature. Early work by Rotter (1967) framed trust as a generalized expectancy, while later research by Mayer et al. (1995) positioned it as a willingness to be vulnerable under conditions of risk and uncertainty. Subsequent scholarship broadened the construct across disciplines, emphasizing its multifaceted and situational nature (Rousseau et al., 1998) before extending it to human-automation contexts (Lee and See, 2004). More recently, efforts to define trust specifically in AI systems have emerged, such as Jacovi et al. (2021)'s notion of contractual trust; however, there remains no consensus in the literature, particularly as generative AI systems introduce social and emotional dimensions that extend beyond purely functional performance (Ng and Zhang, 2025; Sun Q. et al., 2025). This lack of a unified definition makes it difficult to compare findings across studies and limits the development of cumulative knowledge, especially as AI systems become increasingly conversational and relational.

For the purposes of this review, trust in an AI-enabled system is defined as a user's willingness to be vulnerable to the actions of an AI system based on the expectation that the system will perform its intended function (Mayer et al., 1995). This definition is one of the most adopted across both human-computer interaction and organizational research, making it well-suited for synthesizing insights across diverse AI contexts (Bach et al., 2024). Additionally, trust is not a static construct; it evolves over time as users interact with a system, observe its successes and failures, build rapport, and update their expectations accordingly (Akash et al., 2017). This dynamic quality is particularly salient in AI-enabled systems, where probabilistic outputs and shifting system behaviors require users to continuously recalibrate their understanding of system capabilities.

#### 1.1.2 Interaction design factors

Central to this review is the concept of an *interaction design factor*, defined as a design element that can be manipulated to shape how users interact with a system. While Bach et al. examine a broader range of factors that influence trust, this review specifically targets those that are design-oriented in nature. Interaction design factors are operationalized in this study using Crampton Smith's four-dimensional framework (Crampton Smith, 2007). This framework was selected for its breadth and flexibility in accommodating the novel interaction paradigms that AI-enabled systems continue to produce, paradigms that more narrowly defined frameworks may not yet fully capture.

The four dimensions are:

1. **Semantics**: The words and language through which a system communicates, including tone of voice and linguistic framing.
2. **Visual representations**: Non-verbal visual elements such as typography, iconography, diagrams, and visual hierarchy.
3. **Physical embodiment**: The physical objects and spatial configurations through which users interact with a system.
4. **Time**: The dynamic ways in which system behavior and outputs evolve during an interaction.

This framework finds partial support in Nielsen's taxonomy of independent variables in technology research, which includes device type, input modality, and visual design parameters such as font size, contrast, and interface architecture (Lazar et al., 2009). Notably, Nielsen does not treat time as a manipulable design variable, instead positioning it as a dependent measure of interaction efficiency. Crampton Smith's inclusion of time as a design dimension is therefore particularly relevant for AI-enabled systems, whose outputs may evolve adaptively throughout the course of an interaction.

Prior human-computer interaction research has examined a wide range of interaction design factors in AI contexts. Explainable AI (XAI), for instance, has received considerable attention across diverse domains and user populations, motivated by the goal of making AI reasoning more visible so that users can make informed decisions about when and how much to rely on a system (Gade et al., 2019; Dwivedi et al., 2023). Anthropomorphism – the attribution of human characteristics, emotions, or intentions to non-human entities–has also been widely studied, with research examining how human-like design features influence user perception, engagement, and trust (Hai et al., 2026). Together, these lines of research reflect a broader effort within the field to understand how specific design decisions shape the user-AI relationship. Further, an AI system's own projection of confidence in its performance has also been explored as a factor that can influence trust specifically through indicators to users (Bruzzese et al., 2020). Further, these systems have been explored in a variety of modalities from chatbots confined to the computer screen to embodied robots (Wang et al., 2026; Reig et al., 2019).

### 1.2 Methods

#### 1.2.1 Search strategy

This non-registered review followed the Preferred Reporting Items for Systematic Reviews and Meta-Analyses (PRISMA) guidelines (Moher et al., 2010) (see Figure 1). Studies were retrieved from four databases relevant to the field of interaction design: IEEE Xplore, ACM Digital Library, Scopus, and Web of Science. The search for this systematic literature review took place in June of 2025 and its corpus was restricted to publications from January 2023 through June 2025 in order to capture the most current landscape of interaction design research involving AI-enabled systems. Additionally, given the language proficiencies of the research team, only English-language publications were included.

**Figure 1:**
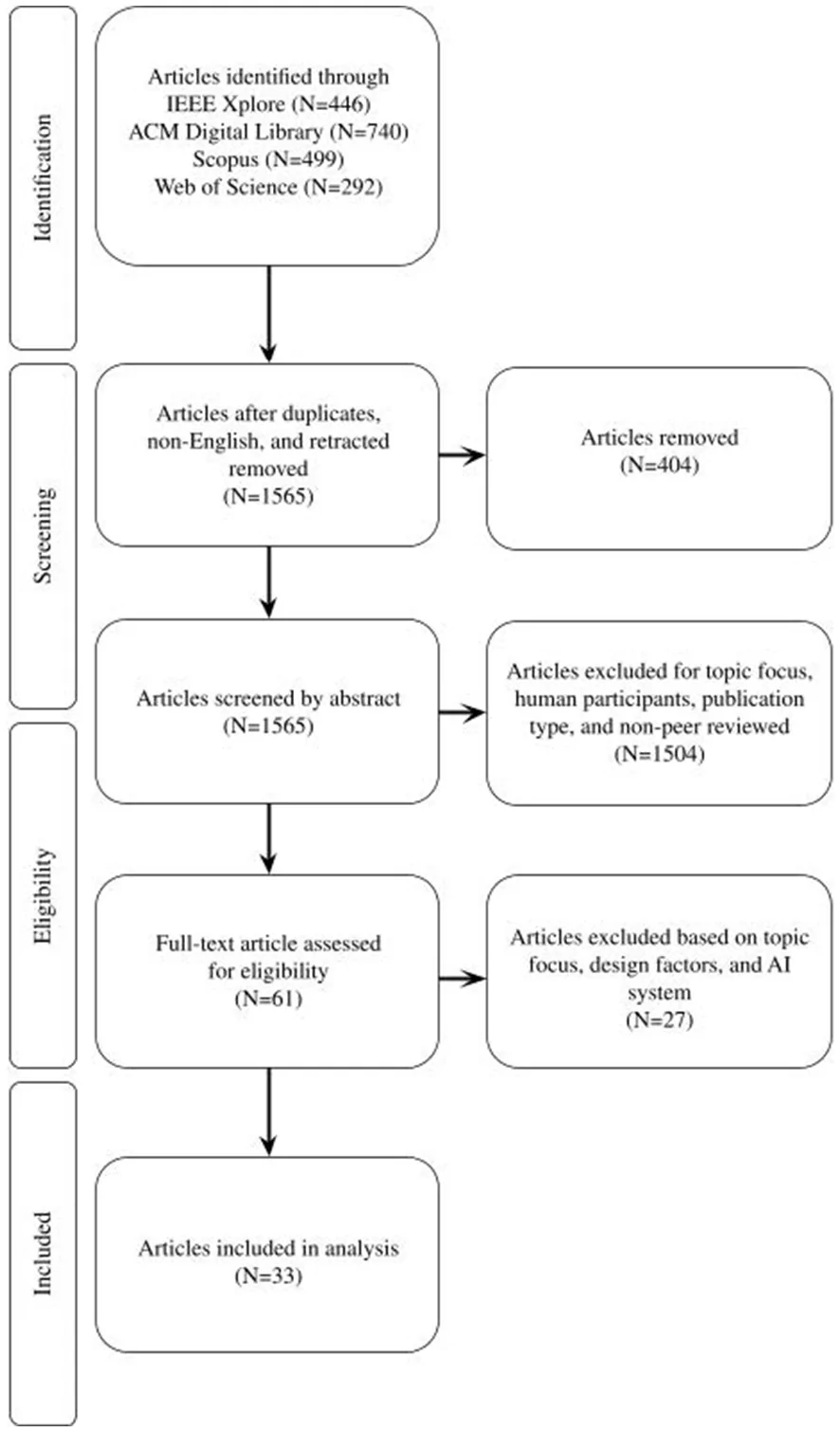
PRISMA flow diagram showing the study identification, screening, eligibility, and inclusion process.

Search terms were developed in consultation with the research questions and an independent librarian. The search strategy was intentionally designed to identify studies examining user trust in AI-enabled systems through an interaction design lens. While RQ1 and RQ2 characterize the broader literature, RQ3 specifically focuses on interaction design factors affecting trust (see Section 1). Accordingly, the search terms combined trust-related concepts with AI and interaction design terms rather than attempting to capture the broader trust-in-AI literature irrespective of design considerations.

Following a pilot test confirming that the selected terms returned all relevant articles while minimizing false positives, the final search string resulted in the terms included in Table 1.

**Table 1:** Search terms used to identify studies examining trust in AI-enabled systems.

Concept	Search terms
Trust Measurement Terms	“trust measurement” OR “trust assessment” OR “trust evaluation” OR “trust metric” OR “trust scale” OR “trust questionnaire” OR “trust survey” OR “trust model” OR “trust framework” OR “measuring trust”
Interaction, Interface Context	“trust in interaction design” OR “trust in user interface” OR “trust in user experience” OR “trust in HCI” OR “trust in human computer interaction” OR “trust in interface design” OR “trust in explainability” OR “trust in transparency” OR “trust in user control” OR “trust in interactivity”
AI System Terms	“artificial intelligence” OR “AI” OR “machine learning” OR “deep learning” OR “neural network” OR “predictive model” OR “explainable AI” OR “XAI” OR “interpretable AI”

All terms were combined using the Boolean operator AND to construct the final search string.

### 1.3 Study selection

Inclusion criteria were developed to directly address the research questions. To be eligible, a study was required to:

1. Be published in English between January 1, 2023 and June 10, 2025;
2. Investigate how interaction design factors (semantic, visual, physical, or time) influence trust (Crampton Smith 2007).
3. Evaluate an AI-enabled system through direct interaction or simulation;
4. Involve human participants;
5. Report empirical findings, thereby excluding opinion pieces and theoretical papers; and
6. Be peer-reviewed.

Consistent with the scope of the review, studies addressing trust in AI, HCI, or human-AI interaction more broadly were not excluded on the basis of disciplinary framing. Rather, they were eligible when they empirically examined the relationship between user trust and at least one interaction design factor. Studies that discussed trust without examining how a design-related characteristic of the AI-enabled system or interaction influenced trust fell outside the scope of the review.

These criteria were applied sequentially across two screening stages. During abstract screening, each abstract was reviewed by one researcher. Straightforward exclusions were made independently, while ambiguous cases were escalated to the full team for a consensus determination.

During full-text screening, one researcher reviewed each paper in full and recorded an inclusion recommendation. The first and second authors then independently reviewed all full-text papers for accuracy, resulting in the exclusion of four additional articles. All authors agreed that the **final 33 articles** satisfied all inclusion criteria.

### 1.4 Data extraction and analysis

The following information was extracted from each included article: year of publication, author names and institutional affiliation, article discipline, definition of trust, AI system evaluated, trust measurement method, sample size and participant demographics, design factor(s) examined, keywords, and an assessment of paper bias/limitations.

#### 1.4.1 Discipline categorization

Articles were categorized by discipline using the Web of Science Research smart search database. Each article title was first searched in the database to identify its designated research area (*n* = 26). For articles not indexed in Web of Science, the journal of publication was searched and its associated research area was assigned (*n* = 5). When neither the title nor the journal could be located, the abstract was read and the research area(s) most closely aligned with the paper's topics and themes were assigned (*n* = 6). Two researchers independently applied this procedure to each article, reaching agreement on 82.35% of cases; disagreements were resolved through consensus.

#### 1.4.2 Design factor thematic analysis

During full-text screening, each reviewer identified the primary interaction design factor manipulated in each paper. These identified factors were then inductively grouped into nine thematic categories by four independent researchers: Explainability, Anthropomorphism, Confidence, Interface, Errors, Security, Transparency, Competence, and Perceived Control. Disagreements were handled through consensus.

This thematic categorization was developed to facilitate systematic comparison between articles that helped to uncover trends in the way these systems are being researched and their findings.

## 2 Results

### 2.1 Author affiliation

Author affiliations were recorded for all included papers. As some papers listed multiple institutional affiliations, the total number of affiliations exceeds the number of included papers. Imperial College London was the most frequently represented institution (n=4, 6.35%), followed by Eindhoven University of Technology, Delft University of Technology (TU Delft), Tilburg University, University of Amsterdam, Southeast University, Tokyo Metropolitan University, Kanagawa University, Shizuoka University, University of Southampton, and University of Colorado Boulder, each appearing twice (n=2, 3.17%). The remaining 37 institutions each contributed a single affiliated author, collectively representing 56.92% of all recorded affiliations. By institution type, the majority of affiliations were from academic institutions in higher education (n=54, 85.71%), while industry and research organizations accounted for the remainder (n=9, 14.28%).

### 2.2 Paper discipline

The included papers spanned a range of research disciplines. Computer science was the most represented area (*n* = 29, 78.38%), followed by engineering (*n* = 7, 18.92%). The remaining papers were distributed across robotics, medical informatics, information science and library science, social sciences, history and philosophy of science, science and technology, business, and art, with each discipline represented by a single paper (*n* = 1, 0.03%). Multiple papers spanned more than one discipline, yielding 46 total disciplinary classifications across the included papers.

### 2.3 Trust definitions

The corpus (n=21, 63.64%) reported a trust definition and the remaining (n=12, 36.36%) did not (Ahn et al., 2024; Alimardani et al., 2024; Feng and Chaspari, 2024; Freel et al., 2025; Kang et al., 2025; Kim et al., 2024; Mühl et al., 2024; Nahulanthran et al., 2025; Nimmo et al., 2024; Robb et al., 2023; Sung et al., 2024; Wang and Yin, 2023).

The most cited definition was (Lee and See, 2004), cited six times, defining trust as “the attitude that an agent will help achieve an individual's goals in a situation characterized by uncertainty and vulnerability.” (Mayer et al., 1995), cited four times, define trust as “the willingness of a party to be vulnerable to the actions of another party based on the expectation that the other will perform a particular action important to the trustor, irrespective of the ability to monitor or control that other party.” Rousseau et al. (1998), cited twice, define trust as “a psychological state comprising the intention to accept vulnerability based upon positive expectations of the intentions or behavior of another.” Table 1 in the Appendix presents the trust definitions used across the reviewed papers and their sources.

Across the 21 papers that reported a trust definition, nine distinct definition sources were identified, indicating a lack of consensus on a single definition of trust within the reviewed literature. Moreover, the most frequently cited sources Lee and See (2004) and Mayer et al. (1995) originate from the fields of automation and organizational psychology, respectively.

### 2.4 Trust measurement

The majority of the studies (*n* = 24, 72.73%) used participants' self-reporting (i.e., self-report) as the only method of trust measurement. This method was followed by mixed methods (*n* = 7, 21.21%), where self-report was combined with other approaches such as behavioral, physiological, or qualitative methods. Behavioral methods alone accounted for (*n* = 2, 6.06%) of the studies. The Appendix includes Table 2, which summarizes the trust measurement methods used across the reviewed studies.

Given that self-report was the predominant trust measurement method, it is relevant to examine the scales and instruments used across the 24 studies that employed this approach (see Table 2 in the Appendix). Hoffman et al. (2018, 2023) was the most frequently cited source, appearing in seven studies (21.21%). Seven additional sources were each cited in two studies (6.06% each), and 15 sources appeared only once (3.03%), reflecting a diversity of measurement instruments. Finally, one study developed its own scale without referencing any external source (Wang and Yin, 2023).

Mixed methods were used in seven studies (21.21%). The most common combination was self-report and behavioral measures, appearing in five studies (Goubard and Demiris, 2025; Mehrotra et al., 2025, 2024b; Wang and Yin, 2023; Kahr et al., 2024). One study combined self-report with qualitative methods (Shukla et al., 2025), and one combined self-report with physiological measures (Sung et al., 2024). Finally, two studies relied exclusively on behavioral measures (Kahr et al., 2023; Ahn et al., 2024).

### 2.5 AI systems measured

The majority of AI systems measured in the literature review corpus include Machine Learning (ML) algorithms, Convolutional Neural Networks (CNNs), Deep Neural Networks (DNNs), Vision-Language Models (VLMs), simulated recordings, simulated prototypes, and conversational AI. Among these categories, simulation-based AI appears the most often (*n* = 13), followed by conversational AI (*n* = 10), ML Algorithms (*n* = 4), and CNNs (*n* = 4). Table 2 presents the distribution of AI enabled systems measured and examples of studies that met the criteria for each system type from the corpus.

**Table 2:** Distribution of AI types and examples (column-width optimized).

AI type	Examples/references
Simulation, Prototype	AI-powered Healthcare Chatbot (Huang and Ki, 2023), AI Classifier System (Freel et al., 2025)
Conversational AI, Large Language Model (LLM)	Voicebot Therapist (Mühl et al., 2024), Chatbot (Elkhatibi et al., 2025)
Neural Network Model (CNN/DNN/VLM)	ResNet-19 with Grad-CAM Heatmap (Maehigashi et al., 2024a), Visual Classification System (Maehigashi et al., 2024b)
ML-Based Decision System	Student Performance Prediction (Ahn et al., 2024), Speech-based Explainable Algorithm (Feng and Chaspari, 2024)
Autonomous Drone	Drone (Cheung and Ho, 2025)
Not Stated	AI evaluated, but system type not specified (Mehrotra et al., 2024b)

Distribution of AI types and examples (column-width optimized).

### 2.6 Population

Population size was recorded for each included research study. Across the literature review corpus, population sizes ranged from a minimum of *n* = 10 to a maximum of *n* = 1,511, with a mean population of *n* = 245.73. For studies conducted in multiple parts utilizing distinct participant groups, population figures were aggregated to reflect the total number of participants surveyed. The distribution of population sizes across the corpus is presented in Figure 2.

**Figure 2:**
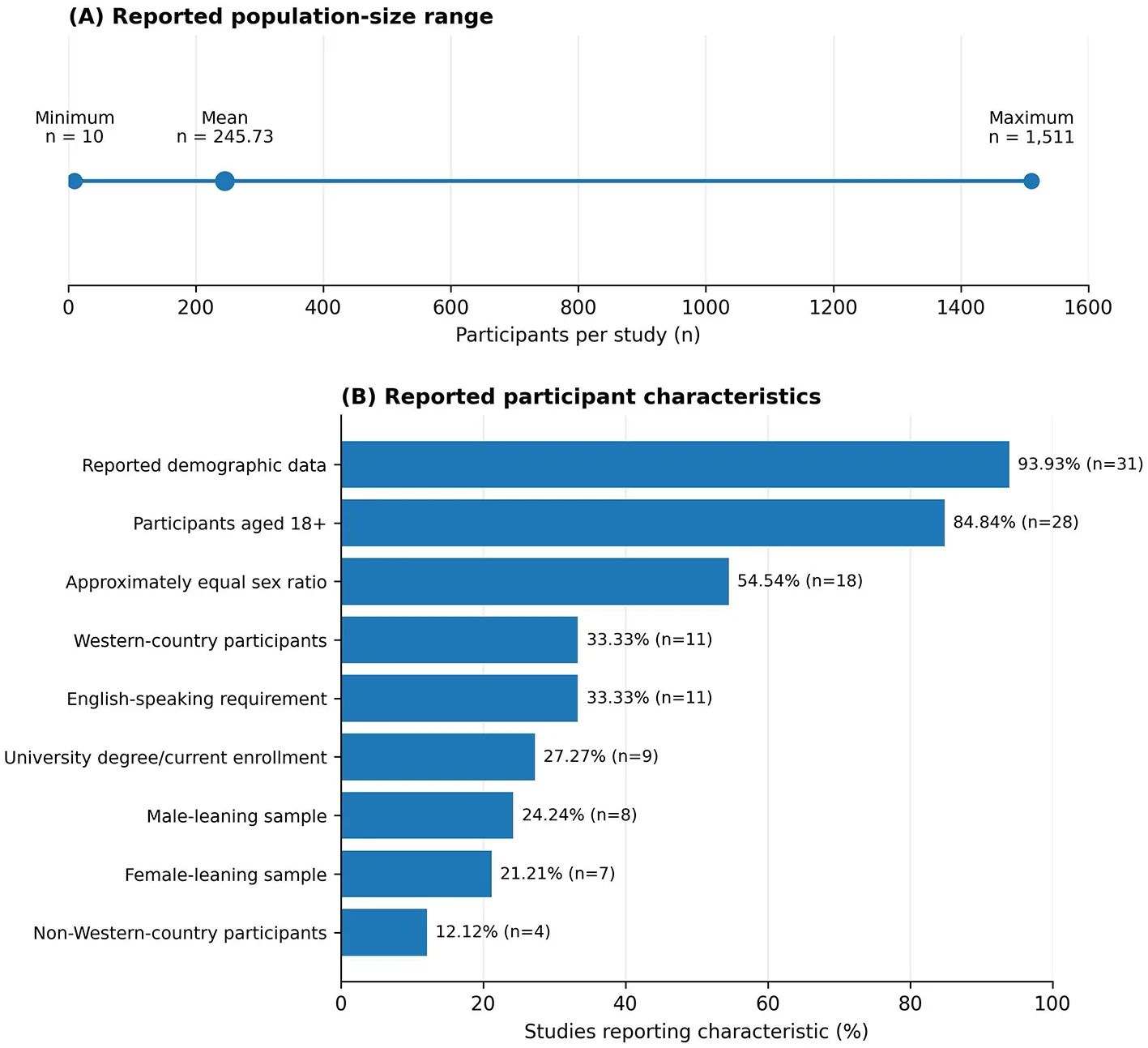
Population characteristics across the 33 reviewed studies. **(A)** presents the reported minimum, mean, and maximum study population sizes. **(B)** summarizes the participant demographic and sampling characteristics reported across the corpus.

Of the corpus, the majority (n=31, 93.93%) reported population demographic data. 18 of the studies (54.54%) reported an approximately equal sex ratio, while (*n* = 8, 24.24%) leaned toward male participants and another (*n* = 7, 21.21%) leaned toward female participants. It may also be noted that (*n* = 28, 84.84%) of studies reported participants aged 18 or older. Regarding geography, (*n* = 11, 33.33%) of studies reported participants from Western countries including Australia, the UK, the US, Canada, and Germany (Widjaya et al., 2023; Nahulanthran et al., 2025; Kang et al., 2025; Krop et al., 2024), with (*n* = 11, 33.33%) of studies imposing an English-speaking requirement. In contrast, only (*n* = 4, 12.12%) of the studies reported participants solely from non-Western countries, including Singapore, South Korea, and Morocco (Cheung and Ho, 2025; Kang et al., 2025; Elkhatibi et al., 2025). Finally, (*n* = 9, 27.27%) of the studies involved participants who had either completed or were currently pursuing a university degree.

### 2.7 Design factors

Design factor categories emerged from the literature review corpus through author consensus, with each paper coded according to the primary interaction design factor examined in relation to user trust. As some studies investigated multiple design features, certain papers appear across more than one category. The distribution of design factors across the corpus is presented in Table 3.

**Table 3:** Counts for AI trust-related factors identified in the reviewed studies.

Factor	Count
Explainability	20
Anthropomorphism	5
Confidence	3
Interface	2
Errors	2
Security	1
Transparency	1
Competence	1
Perceived Control	1

Although Crampton Smith's framework (Crampton Smith, 2007) guided our conceptualization of interaction design factors (see Section 1.1.2), the categories reported in this section were not imposed a priori. Instead, they emerged inductively from the included studies through iterative coding and author consensus. Thus, the nine design factors should be interpreted as recurring manifestations of interaction design decisions situated within Crampton Smith's broader dimensions (Crampton Smith, 2007) rather than as an alternative theoretical taxonomy. Multiple design factors may occupy the same interaction dimension, and some factors span more than one dimension because AI-enabled systems frequently combine linguistic, visual, temporal, and embodied interaction characteristics (see Table 4).

**Table 4:** Relationship between Crampton Smith's four interaction design dimensions and the design factors synthesized from the reviewed literature.

Crampton Smith dimension	Associated design factors
Semantics	Explainability, Confidence, Transparency
Visual Representation	Explainability, Interface, Security
Physical Embodiment	Anthropomorphism, Interface
Time	Errors, Perceived Control, Competence, Explainability

Several design factors span multiple dimensions because AI-enabled systems often combine linguistic, visual, embodied, and temporal interaction characteristics.

#### 2.7.1 Explainability

Studies identified as relating to explainability examined the role of explainable artificial intelligence (XAI) as a design factor across 20 papers, with an average sample size of n=285. The most commonly studied AI systems were Conversational AI (and conversational large language models—LLMs) and Simulation/Prototype models, each represented in five and six studies respectively. Research on XAI employed considerable variation in explanation format, with studies comparing heatmap-based, textual, and multi-format explanation conditions.

Studies reported nuanced findings when manipulating explainability in AI-enabled systems. Several studies found that explainability positively increased user trust and reliance, particularly in complex visual tasks where heatmap-based explanations aided user comprehension of AI reasoning (Maehigashi et al., 2024a,b). One study uniquely found that perceived explainability was positively associated with all three dimensions of trust in AI engineers—ability, benevolence, and integrity—which subsequently influenced the intention to use the AI system (Cheung and Ho, 2025). In contrast, other studies reported that providing explanations, particularly multiple types of explanations simultaneously, increased cognitive load (Maehigashi et al., 2024a), slowed user performance (Maehigashi et al., 2024b), and in some cases did not improve trust even when explanations were present (Mehrotra et al., 2025).

#### 2.7.2 Anthropomorphism

Studies identified as relating to anthropomorphism focused on manipulating human-like qualities such as conversation style (Huang and Ki, 2023), voice (Huang et al., 2024; Alimardani et al., 2024), context framing (Krop et al., 2024), and agent appearance (Huang and Ki, 2023; Alimardani et al., 2024). Four of the anthropomorphism-focused studies utilized factorial experimental design approaches, while one study opted for a long-term use analysis with AI users who had already interacted with the system. The average sample size across these studies was n=197.

The factorial experimental designs manipulating anthropomorphic qualities produced mixed results, with several finding no significant or only conditional effects on trust. Specifically, Alimardani et al. (2024) found that a voice-appearance mismatch did not influence trusting behavior, Kahr et al. (2023) reported no effect of human-like vs. non-human-like explanation types on trust, and Krop et al. (2024) found that humanness framing only influenced advice acceptance when controlling for perceived competence.

In contrast, the long-term analysis study found that respondents who perceived their virtual personal assistant (VPA) as more anthropomorphic reported higher levels of integrity-based trust (Huang et al., 2024), defined as the perceived ability of an AI to make and uphold good-faith agreements (Mayer et al., 1995). Additionally, Huang and Ki (2023) found that the direction of anthropomorphism's effect on trust varied by cue type: human-like warmth generated greater trust than competence in human-like chatbots, whereas competence generated greater trust than warmth in machine-like chatbots.

#### 2.7.3 Confidence

Studies identified as relating to confidence examined how AI systems communicate certainty (Zhang et al., 2020). This design factor was represented by three papers (0.08%), with an average sample size of *n* = 151. Confidence was manipulated across these studies through three distinct approaches: the expression and wording of AI responses, explicit comparison of AI responses to known correct information, and the provision of visual or quantified evidence alongside responses (Sung et al., 2024; Kim et al., 2024; Radensky et al., 2023). All three studies examined confidence through simulated AI-enabled systems, though each employed a different manipulation method. One study manipulated confidence through specific response phrasing, contrasting weaker expressions such as “I'm not sure, but…” against stronger expressions such as “The answer is…” (Kim et al., 2024). A second study operationalized confidence through explicit numerical indicators, exposing participants to the same responses paired with varying internal confidence scores (Sung et al., 2024). A third study constructed experimental conditions in which participants were exposed to AI responses that were clearly correct or incorrect, with and without accompanying express confidence (Radensky et al., 2023).

#### 2.7.4 Interface

Studies identified as relating to interface design examined the effects of manipulating system modality and embodiment. This design factor was represented by two papers, with an average sample size of n=18. The included studies focused on comparing communication channels in healthcare contexts. Notably, four authors appear across the two studies in this group, suggesting a connected line of research rather than independent replication. One study compared text-based, speech, and embodied interfaces, finding that text-based systems were associated with higher trust due to ease of information processing (Sun X. et al., 2025). A related study found that embodied interfaces raised distinct user concerns around privacy and authenticity (Sun et al., 2024). Across both studies, participants demonstrated a preference for text-based conversational user interfaces (CUIs) over speech or embodied formats, attributed to perceptions of reliability.

#### 2.7.5 Errors

Studies identified as relating to errors examined the effects of manipulating error timing and severity on user trust. This design factor was represented by two papers, with an average sample size of n=286. The included studies focused on how the placement of errors within interactions affects trust dynamics. Notably, both studies approached this question from different contextual stakes, offering complementary rather than conflicting perspectives. One study found that errors occurring later in an interaction were more damaging to trust, while the other suggested that early errors had the greater impact (Freel et al., 2025; Kahr et al., 2024). A point of agreement across both, however, was that trust can recover relatively quickly if the system performs well following an error, particularly when users have sufficient post-error experience. Freel et al. (2025) additionally found that the effects of error timing were highly context-dependent: errors in high-stakes systems caused an abrupt collapse of trust, whereas users in low-stakes scenarios were more forgiving, often maintaining trust despite identical errors.

#### 2.7.6 Emerging design factors

Several design factors were represented by a single study each within the corpus, indicating limited research attention and existing gaps in the current literature. These factors included security, transparency, competence, and perceived control:

1. **Security**—Features impacting a users' perceived confidentiality and safety when interacting with an AI-enabled system, examined specifically in the context of AI applications in banking (Elkhatibi et al., 2025).
2. **Transparency**—Design choices related to how an AI system communicates its thought process and decision-making to users, with one study examining the effect of transparent system behavior on trust calibration (Mehrotra et al., 2024b).
3. **Competence**—Refers to the perceived expertise or capability of an AI system in producing accurate and reliable outputs (Krop et al., 2024).
4. **Perceived Control**—The users' perception of their ability to directly influence system behavior, models, or outcomes (Degachi et al., 2023).

## 3 Discussion

### 3.1 RQ1: disciplinary and institutional trends

#### 3.1.1 Author affiliation

Author affiliations spanned 48 institutions, indicating broad interest in user trust in AI-enabled systems. No single institution dominated the corpus; the most represented, Imperial College London, accounted for just four studies (*n* = 4, 6.15%). This diversity suggests the literature reflects multiple perspectives rather than a few dominant research groups. This institutional diversity aligns with the broader pattern observed by Bach et al., whose corpus spanned studies conducted primarily across the USA and Germany, suggesting that while geographic concentration exists, no single institutional center of gravity has emerged to define the field (Bach et al., 2024).

Most studies came from academia (*n* = 54, 85.71%), with a notable minority from industry and research organizations (*n* = 9, 14.28%), including Microsoft, Google, and Nokia Bell Labs, often in collaboration with academic partners. This mix highlights both foundational and applied interest, which may help accelerate the translation of research into real-world system design. Beyond demonstrating broad institutional engagement, these findings suggest that research on trust in AI-enabled systems is characterized by a distributed rather than institutionally concentrated research landscape. Although this diversity may encourage methodological and application-specific innovation, it may also contribute to the conceptual fragmentation observed throughout the literature, as independent research groups adopt different theoretical perspectives, definitions, and measurement approaches. Greater inter-institutional collaboration may therefore help promote shared conceptual frameworks and evaluation practices that facilitate cumulative knowledge building.

#### 3.1.2 Paper discipline

While concentrated in computer science (CS), the literature spans multiple disciplines, including medical informatics, engineering, robotics, social sciences, and even art. This pattern of concentration alongside breadth is consistent with Bach et al. (2024), who found that over half of their included studies focused on robotics and e-commerce domains that similarly reflect a center of gravity in technical disciplines while leaving adjacent fields underrepresented. This breadth reflects both the richness and the distributed nature of perspectives in the field; researchers working within a single discipline risk missing parallel developments, but deep disciplinary expertise remains a genuine asset.

Rather than abandoning that expertise, scholars may benefit from engaging with work beyond their home discipline, treating interdisciplinary awareness and disciplinary depth as complementary orientations rather than competing ones. The interdisciplinary nature of this literature further suggests that future research may benefit from greater theoretical integration across disciplines. Combining perspectives from computer science, psychology, human-computer interaction, and the social sciences may help reconcile the fragmented conceptualizations of trust identified in this review while preserving the contextual insights contributed by each field.

### 3.2 RQ2: definitions, measurement strategies, and populations used to evaluate user trust

#### 3.2.1 Trust definition

From 33 reviewed papers, nine distinct definition sources were identified, revealing a notable lack of consensus on how trust is defined across the reviewed literature. These findings contrast with prior work in a way that suggests a positive trend toward greater definitional transparency. Bach et al. (2024), reviewing studies published between 2011 and 2021, found that only 30.43% of papers explicitly defined trust. Ueno et al. (2022) similarly found that less than half of surveyed papers reported an explicit definition, with one quarter relying solely on Lee and See (2004). The higher rate observed in the present corpus (63.64%) may suggest that the field is gradually moving toward greater definitional transparency. Nevertheless, Mehrotra et al. (2024a) noted that consensus on the definition of appropriate trust remains elusive, with varying perspectives contributing to a lack of clarity in the field. The lack of consensus has implications beyond conceptual clarity. When researchers adopt different theoretical foundations to define trust, comparisons across studies become increasingly challenging, making it more difficult to synthesize findings and establish cumulative knowledge. Greater consistency in how trust is conceptualized would therefore not only strengthen theoretical development but also improve the comparability and reproducibility of future research.

The heterogeneity of AI as a technology adds another layer of complexity to this challenge. Ideally, the field would develop domain-specific conceptualizations of trust that reflect the distinct nature of each AI application, what trust means when interacting with a Large Language Model (LLM) may differ substantially from trust in a diagnostic AI or an autonomous robot, yet the literature has not yet developed definitions that reflect these distinctions. However, given the rapid evolution of AI and the practical challenges of developing and validating definitions for each emerging domain, such convergence remains a theoretical aspiration rather than an immediate possibility. From a pragmatic standpoint, the recommendation advanced by Bach et al. (2024) to select the most appropriate existing definition according to context rather than pursuing a universal one, offers a more realistic path forward for the field in its current state. This perspective also raises an important direction for future research. Rather than seeking a single universal definition applicable to all AI-enabled systems, future work could focus on identifying which dimensions of trust remain consistent across AI applications and which are inherently shaped by the characteristics of a particular domain. Such an approach may help balance theoretical consistency with the contextual differences that characterize modern AI technologies.

Likewise, this reliance on definitions from adjacent fields is evident in the reviewed papers. While these definitions originate from automation and organizational psychology, the reviewed papers show evidence of adaptation to AI contexts. For instance, some papers explicitly modified Lee and See (2004)'s original definition to reference AI agents specifically, as seen in formulations such as “the attitude that an [AI] agent will help achieve an individual's goal in a situation characterized by uncertainty and vulnerability” (Kahr et al., 2023). These adaptations suggest that researchers recognize the need to contextualize borrowed definitions, yet no consensus on an AI-specific definition has emerged. This pattern may reflect the current stage of maturity of the field. Rather than replacing existing conceptualizations of trust, researchers appear to be progressively adapting well-established definitions to better reflect AI-enabled systems. As AI technologies continue to evolve, these incremental refinements may provide the foundation upon which more mature AI-specific conceptualizations of trust can eventually emerge.

The heterogeneity of AI as a technology adds another layer of complexity to the lack of definitional consensus, as trust is also dependent upon application domain. In response to this challenge, some researchers have proposed definitions oriented specifically to AI. Siau and Wang (2018) define trust in AI as the willingness of people to accept AI and believe in the suggestions and decisions made by the system, share tasks, contribute information, and provide support to such technology. More recently, Afroogh et al. (2024) have also conceptualized trust specifically within the context of AI, signaling that AI-specific definitions are beginning to emerge in the literature.

The rapid advancement and diversification of AI further complicates this landscape. AI has evolved through successive paradigms, from machine learning and deep learning, to LLMs, and most recently to autonomous agents, each representing a qualitatively different form of human-AI interaction with distinct implications for how trust is formed, calibrated, and maintained. This trajectory suggests that a single overarching definition of trust becomes increasingly insufficient as the technology diversifies, and that domain-specific conceptualizations will eventually be required. Although such definitional convergence may be gradual, it is anticipated that the AI-specific definitions beginning to emerge in the literature will become more widely known, adopted, and refined over time. In the meantime, the field continues to rely on the foundational definitions that have guided research to date.

Looking ahead, future research should examine whether emerging AI paradigms require new conceptualizations of trust or whether existing theoretical foundations remain applicable across technologies and domains. Comparative studies spanning different AI application domains could help distinguish those aspects of trust that generalize across contexts from those that require domain-specific refinement. Such efforts would contribute to greater conceptual consistency while providing more targeted guidance for the design of trustworthy AI-enabled systems.

#### 3.2.2 Trust measurement

The predominance of self-report as the primary trust measurement strategy across 24 of the 33 reviewed studies (72.73%) reveals a pattern consistent with prior work. Bach et al. (2024) found a near-identical rate of 69.56% across their corpus of 23 studies published between 2011 and 2021, suggesting that this reliance on subjective measurement has persisted into the post-generative AI era. While self-report instruments offer practical advantages, their outcomes can be difficult to compare directly with system trustworthiness levels, and reliance behavior may be driven by factors beyond trust alone (Zhang et al., 2022), suggesting that subjective perception alone is insufficient to capture the full complexity of trust in human-AI interaction.

This measurement fragmentation has direct consequences for the field's ability to compare findings. When studies use different instruments, adapted scales, and ad hoc questionnaires to measure the same construct, direct comparison becomes difficult (Bach et al., 2024; Mehrotra et al., 2024a). This fragmentation is not only a methodological problem but also a disciplinary one. The corpus spans CS, engineering, robotics, and social sciences, and each discipline brings its own measurement conventions. Like the definitions reviewed in the previous section, the measurement strategies used across the corpus reflect a field that continues to borrow and adapt from adjacent fields without converging on shared standards. This diversity reflects both the interdisciplinary nature of trust research and the evolving state of the field. Nonetheless, the absence of shared measurement practices limits the comparability of findings across studies and complicates efforts to establish cumulative evidence regarding the factors that influence user trust in AI-enabled systems.

Despite this measurement fragmentation, the corpus shows evidence of adaptation. Seven studies cited Hoffman et al.'s trust scale for explainable AI (XAI) (Hoffman et al., 2018, 2023), making it the most frequently referenced instrument. Many scholars cited the 2018 preprint rather than the 2023 published version, suggesting the field adopted it before formal peer-reviewed alternatives existed. Beyond individual instruments, five studies combined self-report with behavioral measures (Goubard and Demiris, 2025; Mehrotra et al., 2025, 2024b; Wang and Yin, 2023; Kahr et al., 2024), reflecting a recognition that what users report and what they do could be distinct phenomena. The single study incorporating physiological measurement moves toward objective trust assessment but stops short of inferring trust from biosignals: it validates a task for capturing time-synchronized biosignals, while trust itself is still measured by self-report, with physiological modeling left to future work (Sung et al., 2024). Beyond this corpus, physiological signals have been pursued as a more objective basis for trust assessment than self-report (Gupta et al., 2019; Ajenaghughrure et al., 2020; Chen et al., 2025), although stable models for inferring trust from biosignals have not yet been established. These developments suggest that trust measurement is gradually expanding beyond reliance on self-report alone. Rather than replacing subjective instruments, behavioral and physiological measures may offer complementary perspectives that capture dimensions of trust not directly reflected in users' self-reported perceptions.

The diversity of measurement approaches across the reviewed corpus underscores the need for greater transparency in instrument selection. The growing use of mixed methods is a promising development, as combining self-report with behavioral or qualitative measures allows researchers to capture dimensions of trust that no single instrument can fully reveal, a view similarly advanced by Mehrotra et al. (2024a). Parallel to the emergence of AI-specific trust definitions discussed previously, there are also signs that AI-specific measurement instruments are beginning to emerge. Hoffman et al. (2023)'s scale for XAI represents one of the earliest examples in this corpus, while more recent work has focused on developing instruments specifically for human-AI interaction contexts (McGrath et al., 2025). Future research may therefore benefit from continued validation and adoption of AI-specific trust measures across different application domains. Greater consistency in measurement practices would facilitate comparisons across studies while supporting the development of a more cumulative body of knowledge on trust in AI-enabled systems.

Beyond greater consistency in measurement practices, trust should also be treated as a dynamic construct that may change over time with repeated use of AI-enabled systems. Longitudinal studies could examine how trust evolves across interactions and how this process is influenced by factors such as explainability, anthropomorphism, system performance, and perceived confidence or competence. Combining repeated self-report measures with behavioral data and qualitative methods may also provide a more complete understanding of the relationship between reported trust and actual interaction behavior.

#### 3.2.3 AI system measured

Simulation-based approaches dominate the corpus, reflecting a methodological preference for experimental control. AI systems are inherently non-deterministic therefore through the use of simulations, researchers can systematically manipulate conditions and isolate specific design factors (Park et al., 2026). In an emerging field where foundational causal relationships are still being established, controlled simulation is a methodologically appropriate approach, allowing researchers to build theoretical scaffolding before introducing the complexity of live system variability. However, the prevalence of simulated systems also raises questions about transferability, as findings derived from prototypes may not generalize to the unpredictable, probabilistic outputs of deployed AI systems. Notably, the use of simulated AI is rarely explicitly justified, indicating a gap in methodological transparency. Greater transparency regarding the rationale for selecting simulated rather than deployed AI systems would improve the interpretation of findings and help clarify the contexts in which experimental results are expected to generalize.

The second most common category, conversational AI, reflects both the growing real-world ubiquity of such systems and their inherent versatility (Scherr et al., 2025). Their adaptability across contexts from banking (Elkhatibi et al., 2025) to therapy (Mühl et al., 2024), makes them a natural fit for human-centered research, and their capacity to simulate social interaction renders trust a particularly salient outcome variable (Smith et al., 2025). ML-based decision systems and neural network models each appear in only four studies, likely reflecting the difficulty of designing human-centered trust paradigms around opaque algorithmic outputs (Adlung et al., 2021). The near-absence of autonomous systems, represented by a single drone study (Cheung and Ho, 2025), is a notable gap given their growing real-world deployment. As AI systems continue to evolve toward greater autonomy, understanding how users develop, calibrate, and maintain trust in these settings represents an increasingly important direction for future research.

Overall, the distribution of AI system types across the corpus suggests that human-centered trust research remains concentrated around systems that afford direct interaction, leaving a substantial portion of the deployed AI landscape underexplored. Future work may therefore benefit from examining trust across a broader range of AI technologies, particularly autonomous and adaptive systems, to determine whether existing theoretical and design recommendations generalize across interaction paradigms or require domain-specific refinement.

#### 3.2.4 Population

These demographic patterns point toward a systematic bias in participant recruitment, consistent with the WEIRD framework (i.e., Western, Educated, Industrialized, Rich, and Democratic) (Henrich et al., 2010). This bias may result in theories derived from a small and unrepresentative segment of the population being generalized more broadly, thereby limiting the external validity of the reviewed studies (Call et al., 2023). The predominance of adult participants in the literature likely reflects the ethical uncertainties and heightened scrutiny associated with conducting digital research with minors. Stricter safeguards around consent, data handling, and risk mitigation for vulnerable populations can limit the inclusion of minors, often precluding them from important research opportunities (Facca et al., 2020).

The geographic skew toward Western countries and limited representation of non-Western populations may have been further reinforced by our restriction to English-language publications, which may have excluded relevant research published in other languages. As noted by Peck et al. (2021) in the context of virtual reality (VR) research, recruitment based on university students, although convenient and common in Human-Computer Interaction (HCI) research, poses limitations to the generalizability of findings due to narrow age ranges and relatively homogeneous educational backgrounds. The high proportion of university-educated participants reflects reliance on easily accessible samples, such as students, which reinforces the WEIRD bias. Taken together, these patterns highlight the need to justify demographic choices and broaden participant diversity in future human-centered AI research.

Importantly, greater demographic and cultural diversity may also affect how the design factors identified in this review relate to trust. The influence of anthropomorphic cues, for example, may vary depending on culturally shaped expectations regarding social interaction, agency, and appropriate human-like behavior from technological systems. Similarly, perceptions of AI competence and expressions of system confidence may be interpreted differently across cultural contexts, potentially affecting when such cues strengthen or undermine trust. Explainability may likewise be evaluated differently depending on users' expectations regarding transparency, authority, and the amount or type of information needed to assess an AI system. The current literature does not provide sufficient cross-cultural evidence to determine whether these relationships differ systematically across populations. Future research should therefore examine these design factors across culturally diverse samples and contexts rather than assuming that their effects on trust generalize uniformly across populations.

### 3.3 RQ3: interaction design factors shown to affect user trust in AI-enabled systems

#### 3.3.1 Design factors

Overall, in terms of manipulating trust through design factors, there is a lack of long-term studies examining the evolution of trust in systems after prolonged interaction (Radensky et al., 2023). Lucas et al. (2014) identified that users who believed they interacted with a computer-based agent, compared to those who believed they interacted with a human operator, were observed more willing to self-disclose and display more openly their emotions. Given the dynamic relationship that develops between users and technology over extended periods of time, future work in this emerging field would benefit from further examining design factors beyond single experimental sessions (Radensky et al., 2023).

Design factors could include interaction dynamics, such as virtual agents' responsiveness during user-agent interactions, upfront transparency on data privacy and usage, or even establishing a virtual agent with a reputable organization (You et al., 2022). Similarly, these factors could also concern user-agent and user-designer synchrony in human-agent interactions (Feijóo-Garćıa et al., 2024), and the interplay among users' demographics and those characteristics posed by virtual agents designed to assist in different contexts (Feijóo-Garćıa et al., 2023, 2024; Ghosh et al., 2023).

Future research should further explore how design decisions, contextual factors, and user characteristics interplay and influence trust in human-agent interactions. Particular attention should be given to longitudinal settings, considering that user may build relationships with agents over time (Bickmore and Picard, 2005), and that agent relationships could evolve over time and shape perceptions, attitudes, behaviors, and engagement. Such work could help contribute design guidelines for developing effective and trustworthy virtual agents for different application domains.

#### 3.3.2 Explainability

There is a plethora of knowledge that has been generated in the post generative-AI area regarding demystifying the AI black-box through explainability (Rai, 2020). Despite how common XAI research is, its influence on trust is complex. Common patterns emerged from the corpus including the accuracy of AI responses having positive correlation with the user's trust in the AI system (Kang et al., 2025; Mehrotra et al., 2024b; Cheung and Ho, 2025; Maehigashi et al., 2024a). However, due to the vast nature of variables that can be manipulated in an AI's response/output there should be an emphasis on facilitating the appropriate amount of trust rather than just increased trust, which is currently not prioritized in the literature (Mehrotra et al., 2025). As AI systems become increasingly accurate, understanding how to foster appropriate levels of user trust will be important. However, given that AI still produces errors and occasional hallucinations, studying trust calibration–ensuring users rely on AI appropriately–is currently the most practical and valuable focus (King, 2025).

Future research should therefore examine which forms of explanation support calibrated reliance under varying levels of system accuracy. Rather than evaluating explanations primarily by whether they increase trust, studies may benefit from assessing whether they help users distinguish between reliable and unreliable system outputs. Comparative investigations of explanation format, timing, detail, and accuracy could clarify when explainability supports appropriate reliance and when it instead contributes to overtrust or unnecessary distrust.

#### 3.3.3 Anthropomorphism

Post-generative AI studies suggest that manipulating anthropomorphism does not produce a clear causal effect on trust in controlled environments, which is contrary to the existing idea that anthropomorphism could enhance trust (Schmitt, 2019). However, this does not preclude its influence, particularly over longer-term interactions where human-like characteristics may shape user perceptions more gradually. Many studies assume that design manipulations inherently evoke anthropomorphic qualities simply because a system appears more human-like. Recent work challenges this assumption by distinguishing between anthropomorphism, the attribution of human characteristics to nonhuman agents, and anthropomorphic response, which captures users' perceptions and behavioral reactions during interaction (Kim and Im, 2023). This distinction highlights the need to evaluate perceived humanness directly, suggesting that measures of anthropomorphic response should be considered alongside trust when assessing the impact of anthropomorphic design.

Future research should therefore distinguish more clearly between the presence of anthropomorphic design features and users' actual perceptions of those features. Longitudinal studies may be particularly valuable for determining whether anthropomorphic responses develop over time and whether their relationship with trust changes as users gain experience with an AI system. Such work could clarify whether anthropomorphism influences trust directly or through related perceptions, such as familiarity, social presence, competence, or perceived intentionality.

#### 3.3.4 Confidence

The studies manipulating confidence as a design factor present nuanced implications. Rather than supporting direct claims that altering a system's confidence signaling uniformly increases or decreases user trust, the findings point to a more conditional relationship. When viewed through the lens of trust calibration, a clearer pattern emerges: confidence signals influence how users distribute their reliance on a system. Misalignment between expressed confidence and actual accuracy tends to produce either over-reliance or degraded trust, whereas well-calibrated expressions of uncertainty support more appropriate reliance decisions (Radensky et al., 2023; Sung et al., 2024; Kim et al., 2024).

In addition, there is limited distinction among the different ways in which system confidence can be communicated. Across the reviewed studies, confidence was manipulated through approaches such as linguistic and numeric cues, yet no direct comparisons were conducted to determine their relative effectiveness. Future research should therefore compare confidence-signaling approaches under equivalent conditions, examining not only their effects on trust but also their ability to support accurate reliance decisions. Replication of existing findings across tasks, populations, and application domains would also help determine whether the observed effects generalize beyond the specific experimental contexts in which they were identified.

Further, this exploration is limited by the sole reliance on simulated AI systems, which constrains its application to real-world scenarios. Studies involving deployed or operational AI systems are therefore needed to determine whether confidence signals remain effective when users encounter naturally occurring uncertainty, variable performance, and repeated interactions. Such work would help establish whether findings derived from controlled simulations transfer to more complex human-AI interaction settings.

#### 3.3.5 Interface

The included studies indicate that users generally exhibit greater trust toward text-based conversational interfaces than toward embodied or voice-based alternatives, particularly in healthcare contexts (Sun X. et al., 2025; Sun et al., 2024). However, the evidentiary strength of these conclusions is limited by the provenance of the studies. Both papers share four authors and originate from the same institution, meaning that the observed patterns cannot be considered independent corroboration. Consequently, the findings reflect a single research program rather than a convergent body of evidence. Until these results are replicated by independent research teams, across diverse methodologies and application contexts, claims regarding the trust-diminishing effects of embodiment should be interpreted cautiously.

This limitation is further highlighted by the findings of Rahman (2024), in which physical embodiment was associated with increased user trust in more casual settings. While these studies present contradictory findings, they also suggest that the relationship between interface embodiment and trust may depend on the deployment context. In clinical or information-intensive settings, concerns regarding surveillance and authenticity may favor disembodied interfaces, whereas in domestic or casual environments, physical presence may enhance perceptions of familiarity and approachability. Future research should explicitly manipulate deployment context as an independent variable to clarify whether observed trust effects are attributable to interface modality itself or to the interaction between modality and environment.

Future studies may also benefit from examining individual interface characteristics, such as voice, visual representation, and physical presence, rather than treating embodiment as a single design factor. Previous work has highlighted the importance of considering these characteristics during the design process and their potential influence on user perceptions (Feijóo-Garćıa et al., 2024, 2023). Building on these efforts, future research could examine how individual interface characteristics, and their combinations, influence trust across different application domains and deployment contexts.

#### 3.3.6 Errors

The limited research on error timing as a manipulable design factor underscores the need for further studies, particularly those examining how timing effects evolve over extended interactions. The contrasting findings across existing studies suggest that the impact of error timing is highly context-dependent, varying with interaction stakes, error severity, and user expectations (Freel et al., 2025; Kahr et al., 2024). Future work should therefore move beyond isolating timing effects and instead examine how timing interacts with contextual factors and post-error system performance. In particular, the observed potential for trust recovery highlights the importance of investigating recovery strategies and the role of continued positive system behavior in mitigating trust loss. Additionally, repeated exposure to errors over time may lead to phenomena such as AI error blindness, in which users become desensitized to mistakes, with important implications for trust calibration and system design (Logg et al., 2019). Understanding how these dynamics unfold longitudinally will be critical for designing systems that maintain appropriate levels of user trust.

Future research should also distinguish among different error types and levels of severity, as factual, procedural, and safety-related errors may produce different patterns of trust loss and recovery. Comparative studies examining strategies such as error acknowledgment, explanation, correction, and subsequent system performance could help determine which approaches most effectively support trust recovery under different conditions.

#### 3.3.7 Emerging design factors

Security, transparency, competence, and perceived control each appear in only a single study, indicating limited exploration and making it difficult to identify consistent patterns. While these factors show potential in shaping trust, the evidence is too sparse to draw definitive conclusions:

1. **Security:** May reduce perceived vulnerability and support trust, but it is unclear which design elements most effectively enhance safety (Elkhatibi et al., 2025).
2. **Transparency:** Helps users assess system behavior, yet alternative mechanisms beyond explanations remain largely unexplored (Mehrotra et al., 2024b).
3. **Competence:** Signals system expertise and credibility, but alignment between perceived and actual capability is not well understood (Krop et al., 2024).
4. **Perceived Control:** Offers users a sense of influence over outcomes, though which interaction features most effectively foster this perception remains unknown (Degachi et al., 2023).

Collectively, these emerging factors suggest multiple avenues for supporting trust, but further research is needed to replicate findings, isolate underlying mechanisms, and examine their effects across contexts and over time. Given the limited evidence currently available, future work should also determine whether these factors operate independently or in combination, and whether their influence varies according to AI application domain, interaction modality, or user population. Such efforts may help establish whether these emerging factors represent distinct dimensions of trust or broader design principles that extend across AI-enabled systems.

### 3.4 Limitations

Several limitations of this study warrant acknowledgment. First, there may have been bias introduced during abstract screening, as each paper was not reviewed by more than one researcher. This single-reviewer process raises the possibility that relevant papers were inadvertently filtered out. However, given the volume of abstracts retrieved in our original search, this caveat was a necessary methodological concession. We believe many potential biases were mitigated through the application of a strict inclusion and exclusion protocol, whereby only papers that were clearly excludable based on our established criteria were removed by a single researcher.

Second, we acknowledge that while our search strings were intentionally constructed to remain broad enough to capture a variety of design factors, they nonetheless reflect a degree of bias toward design factors previously familiar to our research team. As a result, more niche or emergent factors may have been underrepresented in the final corpus.

Finally, by design, this research surveys studies across many disciplines and contexts within Human-Computer Interaction (HCI), and each context carries its own unique implications for community-specific artifacts. A cross-disciplinary comparison of these artifacts will therefore never be perfectly equivalent. We accept this as an inherent limitation of the scope and derive our identified patterns accordingly, with appropriate interpretive caution.

## 4 Conclusion and future directions

This systematic literature review set out to examine three questions: how disciplinary and institutional trends have shaped HCI research on user trust in AI-enabled systems (RQ1); what definitions and measurement strategies characterize that research (RQ2); and what interaction design factors have been shown to influence user trust (RQ3). Across all three, the review surfaces meaningful progress alongside structural challenges that are characteristic of a rapidly evolving field. This systematic literature review reveals that more recent studies of trust and the effects of interaction design on AI-enabled systems are, overall, asking increasingly powerful and specific questions that contribute to a growing understanding of how to design better systems. However, these studies often lack cohesive language and methodological consistency, which limits the development of a more coherent body of literature.

While the field benefits from a diversity of perspectives–drawing from multiple institutions, disciplines, and contexts–this breadth has also resulted in a wide net being cast in many directions. As a result, although a range of contexts and design factors are explored, this fragmentation hinders the field's ability to build upon prior work in a meaningful and cumulative way. This issue is further exacerbated by a lack of methodological coherence, which limits comparability across studies, particularly for emerging or less-explored design factors.

An exception to this trend is the explainability design factor, where a substantial body of literature has enabled clearer comparison and more consistent knowledge building. However, this highlights a challenge for newer design factors that are still in the process of establishing their foundational literature.

From this review, authors should recognize emerging commonalities in methodological approaches, particularly in the adoption of definitions of trust. Specifically, researchers tend to draw on either Lee and See's definition—“the attitude that an agent will help achieve an individual's goals in a situation characterized by uncertainty and vulnerability.” Lee and See (2004)—or Mayer et al.'s definition—“the willingness of a party to be vulnerable to the actions of another party based on the expectation that the other will perform a particular action important to the trustor, irrespective of the ability to monitor or control that other party.” Mayer et al. (1995)—depending on what best fits their experimental context. Greater consistency in such foundational choices would support clearer comparison across studies.

Additionally, the literature would benefit from increased experimentation with real AI systems rather than relying solely on simulations. While both approaches are valuable for building knowledge, simulations can only approximate real-world deployment, limiting the ecological validity of findings. Future research should also prioritize longer-term studies, given the evolving nature of trust over time, building on dimensions such as those proposed by Crampton Smith (Crampton Smith, 2007).

A further limitation identified in this review is the heavy reliance on solely self-reported measures of trust, which poses a significant barrier to the field. While self-report methods provide valuable participant insight, they are often insufficient in capturing the complexity and dynamic nature of trust. Future research should therefore move toward more mixed-method approaches, incorporating behavioral, physiological, psychophysical, and longitudinal measures to provide a more comprehensive and robust understanding of trust in AI systems.

Furthermore, researchers should consider the importance of diverse participant populations. There is currently a lack of representation across ethnicities, ages, education levels, nationalities, and abilities. When designing technologies intended for general public use, it is essential to evaluate them with populations that reflect this diversity. This can be addressed through more inclusive and varied recruitment strategies.

Collectively, these directions are necessary to support the identification of design trends both within specific contexts and across contexts. Advancing in this way will enable the development of more generalizable guidance for understanding and designing AI-enabled systems that foster appropriate levels of trust in users.
