# Supplementary material for: Trust and interaction design in AI-enabled systems: a systematic literature review
Source: Front Psychol. 2026 Sep 15;17:1932655. doi: 10.3389/fpsyg.2026.1932655 (PMC13619576; doi:10.3389/fpsyg.2026.1932655)
Supplement: Supplementary file 1 [file Supplementary_file_1.pdf]

Supplementary Material

1 APPENDIX

Table S1: Trust Definitions Reported in Reviewed Papers

Reviewed Paper	Trust Definition Used	Definition Source
Maehigashi et al. Maehigashi et al. (2024a)	"the attitude that an agent will help achieve an individual's goals in a situation characterized by uncertainty and vulnerability"	Lee & See Lee and See (2004)
Maehigashi et al. Maehigashi et al. (2024b)	"the attitude that an agent will help achieve an individual's goals in a situation characterized by uncertainty and vulnerability"	Lee & See Lee and See (2004)
Mehrotra et al. Mehrotra et al. (2025)	"the belief that an agent will help achieve an individual's goal in a situation characterized by uncertainty and vulnerability"	Lee & See Lee and See (2004)
Radensky et al. Radensky et al. (2023)	"the attitude that an agent will help achieve an individual's goals in a situation characterized by uncertainty and vulnerability"	Lee & See Lee and See (2004)
Kahr et al. Kahr et al. (2023)	"the attitude that an [AI] agent will help achieve an individual's goal in a situation characterized by uncertainty and vulnerability"	Lee & See Lee and See (2004)
Degachi et al. Degachi et al. (2023)	"[the belief] that an agent will help achieve an individual's goal in a situation characterized by uncertainty and vulnerability"	Lee & See Lee and See (2004)
Sun et al. Sun et al. (2024a)	"the user's belief in the system's reliability and credibility, and ability to perform tasks effectively"	Mayer et al. Mayer et al. (1995)
Huang et al. Huang et al. (2024)	"consumers' expectations of positive outcomes performed by a VPA"	Mayer et al. Mayer et al. (1995)
Elkhatibi et al. Elkhatibi et al. (2025)	"The readiness of one party to be vulnerable to the actions of another..."	Mayer et al. Mayer et al. (1995)
Sun et al. Sun et al. (2025)	"the willingness to be vulnerable based on expectations of another's behavior"	Mayer et al. Mayer et al. (1995)
Freel et al. Freel et al. (2025)	"a psychological state comprising the intention to accept vulnerability..."	Rousseau et al. Rousseau et al. (1998)
Shukla et al. Shukla et al. (2025)	"willingness to be vulnerable, a confident and positive expectation..."	Rousseau et al. Rousseau et al. (1998)
Shin DongheeShin (2021)	"a dynamic attitude that is continually calibrated..."	Rempel et al. Rempel et al. (1985)
Smith & ZangSmith and Zhang (2025)	"a dynamic attitude that is continually calibrated..."	Rempel et al. Rempel et al. (1985)

Krop et al. Krop et al. (2024)	"the willingness to rely on and be vulnerable to another party"	Ulfert et al. Ulfert et al. (2023)
Cheung & Ho Cheung and Ho (2025)	"a mental state where the trustor holds an expectation..."	Chen Chen (2021)
Goubard & Demiris Goubard and Demiris (2025)	"a fundamental aspect of human behaviour..."	Boudreau et al. Boudreau et al. (2008)
Pafla et al. Pafla et al. (2024a)	"conceptualized as an attitude rooted in vulnerability..."	Vereschak et al. Vereschak et al. (2021)
Widjaya et al. Widjaya et al. (2023)	"Trust in digital information indicates a positive..."	Rowley & Johnson Rowley and Johnson (2013)
Kahr et al. Kahr et al. (2024)	"cognitive processes and the resulting manifestation..."	Hardin Hardin (2002)
Huang & Ki Huang and Ki (2023)	"the belief in the reliability and trustworthiness..."	Shin Shin (2021)

Table S2: Trust Measurement Reported in Reviewed Papers

**Paper Authors**	**Trust Measurement Strategy**	**Measurement Source**
Krop et al. Krop et al. (2024)	Self-report, Human-Computer Trust Scale Madsen and Gregor (2000), 5-point Likert scale.	Madsen & Gregor Madsen and Gregor (2000)
Cheung & Ho Cheung and Ho (2025)	Trust in AI engineers: Self-report, 3 dimensions (ability, benevolence, integrity), 4 items per dimension, Likert 5 points Mayer and Davis (1999). Trust in AI system: Self-report, 3 dimensions (performance, purpose, process), 4 items per dimension, 5-point Likert scale Jensen et al. (2020).	Mayer & Davis Mayer and Davis (1999); Jensen et al. Jensen et al. (2020)
Goubard & Demiris Goubard and Demiris (2025)	TPS-HRI: Self-report, 14 items, Likert-like (0–100%) Schaefer (2016). MDMTv2: Self-report, 5 dimensions, 5 items per dimension, scale 0–5 Malle and Ullman (2021). Behavioral measure: Binary intervention decision.	Schaefer Schaefer (2016); Malle & Ullman Malle and Ullman (2021)
Freel et al. Freel et al. (2025)	Human-Computer Trust Scale: Self-report Gulati et al. (2019), Cronbach's $\alpha > 0.84$. Qualitative: 2 open-ended questions.	Gulati et al. Gulati et al. (2019)
Maehigashi et al. Maehigashi et al. (2024a)	Trust scale for XAI: Self-report, 8 items, 5-point scale Hoffman et al. (2023).	Hoffman et al. Hoffman et al. (2023)

Maehigashi et al. Maehigashi et al. (2024b)	Trust scale for XAI: Self-report, 8 items, 5-point scale Hoffman et al. (2023).	Hoffman et al. Hoffman et al. (2023)
Mehrotra et al. Mehrotra et al. (2025)	Global Trust Meter: Self-report, continuous slider (-100 to $+100$) Khasawneh et al. (2003) Mehrotra et al. (2024) Yang et al. (2020). Appropriate trust (App1): Behavioral Yang et al. (2020). Appropriate trust (App2): Behavioral Mehrotra et al. (2023). Calibrated trust (Calib1): Behavioral, switch percentage Zhang et al. (2020). Calibrated trust (Calib2): Behavioral, agreement percentage Lai and Tan (2019).	Khasawneh et al. Khasawneh et al. (2003); Mehrotra et al. Mehrotra et al. (2024); Yang et al. Yang et al. (2020); Mehrotra et al. Mehrotra et al. (2023); Zhang et al. Zhang et al. (2020); Lai & Tan Lai and Tan (2019)
Radensky et al. Radensky et al. (2023)	Trust: 6 single-item measures, 7-point Likert scale Tsai et al. (2021), Rader et al. (2018) Balog and Radlinski (2020), Toader et al. (2019).	Tsai et al. Tsai et al. (2021); Rader et al. Rader et al. (2018); Balog & Radlinski Balog and Radlinski (2020); Toader et al. Toader et al. (2019)
Kahr et al. Kahr et al. (2023)	Weight on Advice (WoA): Behavioral, degree to which participants rely on AI advice. Judge-Advisor System paradigm Sniezek and Van Swol (2001).	Sniezek & Van Swol Sniezek and Van Swol (2001)
Degachi et al. Degachi et al. (2023)	Human-Computer Trust Scale: Self-report, 12 items, 5-point Likert scale, 3 dimensions Gulati et al. (2019).	Gulati et al. Gulati et al. (2019)
Mehrotra et al. Mehrotra et al. (2024)	Cognitive trust: Self-report, single binary item Johnson and Grayson (2005). Global Trust Meter: Self-report, linear slider (0 to +100) Khasawneh et al. (2003). Appropriate trust: Behavioral Ferreira Gomes Centeio Jorge et al. (2021).	Johnson & Grayson Johnson and Grayson (2005); Khasawneh et al. Khasawneh et al. (2003); Ferreira Gomes Centeio Jorge et al. Ferreira Gomes Centeio Jorge et al. (2021)

Smith & Zang Smith and Zhang (2025)	Perceived Trustworthiness Survey: Self-report, 7 items, scale 0–100%, 2 dimensions Lee and See (2004).	Lee & See Lee and See (2004)
Sun et al. Sun et al. (2024a)	Trust in technology (pre-study): Self-report, 6 items, 5-point Likert Jessup et al. (2019). Trust in health information (post-task): Self-report, 11 items, 3 dimensions (credibility, usefulness, readability) Rowley et al. (2015) Johnson et al. (2015). Trust in interface (post-task): Self-report, 15 items, adapted from Corritore et al. Corritore et al. (2007).	Jessup et al. Jessup et al. (2019); Rowley et al. Rowley et al. (2015); Corritore et al. Corritore et al. (2007)
Huang et al. Huang et al. (2024)	Trust (Competence and Integrity): Self-report, 8 items, Likert-type Chattaraman et al. (2019) Mayer and Davis (1999).	Chattaraman et al. Chattaraman et al. (2019); Mayer & Davis Mayer and Davis (1999)
Feng & Chaspari Feng and Chaspari (2024)	Merit Scale: Self-report, 6 items, 5-point Likert Hoffman et al. (2018).	Hoffman et al. Hoffman et al. (2018)
Alimardani et al. Alimardani et al. (2024)	Trust questionnaire: Self-report, 12 items, 5-point Likert Jian et al. (2000).	Jian et al. Jian et al. (2000)
Nahulanthran et al. Nahulanthran et al. (2025)	Trust Scale and Explanation Satisfaction Scale: Self-report Hoffman et al. (2018), no items or scale points specified.	Hoffman et al. Hoffman et al. (2018)
Kang et al. Kang et al. (2025)	Trust Scale for Explainable AI (TXAI): Self-report, 8 items, 5-point scale, Cronbach's $\alpha = .84$ Hoffman et al. (2023).	Hoffman et al. Hoffman et al. (2023)
Robb et al. Robb et al. (2023)	Letras-G Trust Scale: Self-report, 7 items Lim et al. (2022).	Lim et al. Lim et al. (2022)
Wang & Yin Wang et al. (2024)	Self-reported trust metric: Self-report, 5 items, 5-point Likert, items 1–3 Shin (2021), items 4–5 Sun et al. (2024b). Behaviour-based trust metric: Behavioral, frequency of appropriate decisions.	Shin Shin (2021)
Ahn et al. Pafla et al. (2024b)	Weight of Advice (WoA): Behavioral, degree to which participants update their response toward the AI's prediction.	—
Mühl et al. Mühl et al. (2024)	Social Trust in Therapy (SV-TAB): Self-report, 12 items, 5-point Likert Hewig et al. (2011). Internet Privacy Concerns (IPC) Scale: Self-report, 14 items, 5-point Likert, 4 subscales Hong and Thong (2013).	Hewig et al. Hewig et al. (2011)

Nimmo et al. Nimmo et al. (2024)	Trust Scale for XAI: Self-report Hoffman et al. (2018).	Hoffman et al. Hoffman et al. (2018)
Wang & Yin Wang and Yin (2023)	Subjective trust and satisfaction (own scale): Self-report, 7-point Likert scale, administered twice (pre and post).	—
Pafla et al. Pafla et al. (2024b)	Satisfaction scale: Self-report, 8 items, 5-point Likert Hoffman et al. (2018). Trust in AI scale: Self-report, 8 items (item 5 excluded), 5-point Likert Hoffman et al. (2018).	Hoffman et al. Hoffman et al. (2018)
Elkhatibi et al. Elkhatibi et al. (2025)	Trust scale: Self-report, 7 items, 5-point Likert, 3 dimensions: Ability (2 items), Benevolence (2 items), Integrity (3 items) Mayer et al. (1995).	Mayer et al. Mayer et al. (1995)
Sun et al. Sun et al. (2025)	Propensity of Trust in Technology Scale: Self-report, 6 items, 5-point Likert Jessup et al. (2019). Trust of Online Health Information: Self-report, 11 items, 5-point Likert Rowley et al. (2015). Model of Online Trust of Health Information Websites: Self-report, 15 items, 5-point Likert Corritore et al. (2007).	Jessup et al. Jessup et al. (2019); Rowley et al. Rowley et al. (2015); Corritore et al. Corritore et al. (2007)
Shukla et al. Shukla et al. (2025)	Rotter Interpersonal Trust Scale + Social Trust Scale: Self-report, 28 items total. Semi-structured interview: Qualitative, 21 respondents, constructivist grounded theory.	Rotter Rotter (1967)
Widjaya et al. Widjaya et al. (2023)	Trust in Automation Scale: Self-report, 19 items, 6 subscales Körber (2019).	Korber Körber (2019)
Kahr et al. Kahr et al. (2024)	Trust (Faith subscale, per trial): Self-report, 3 items per round Madsen and Gregor (2000). Trust (Perceived Reliability and Technical Competence, post-experiment): Self-report, 9 items, Cronbach's $\alpha = 0.93$ Madsen and Gregor (2000). Weight on Advice (WoA): Behavioral Sniezek and Buckley (1995).	Madsen & Gregor Madsen and Gregor (2000); Sniezek & Buckley Sniezek and Buckley (1995)
Sung et al. Sung et al. (2024)	Trust sliders: Self-report, single item, scale "Not at all" to "Completely", administered every 45 seconds. Trust in Automated Systems (TAS) Survey: Self-report Jian et al. (2000). Biosignal sensor streams: Physiological, 6 sensor streams.	Jian et al. Jian et al. (2000)
Huang & Ki Huang and Ki (2023)	Trust in chatbot: Self-report, 6 items Fogg and Tseng (1999) Chen (2021).	Fogg & Tseng Fogg and Tseng (1999)
Kim et al. Kim et al. (2024)	TrustBelief: Self-report, 6 items McKnight et al. (2002). TrustIntention: Self-report, 4 items McKnight et al. (2002).	McKnight et al. McKnight et al. (2002)
